# Transcriptomic Insights into lncRNA–miRNA–mRNA Networks Regulating Angiogenesis and Metastasis in Prostate Cancer

**DOI:** 10.3390/biotech15010012

**Published:** 2026-02-01

**Authors:** Jonathan Puente-Rivera, Stephanie I. Nuñez Olvera, Ameyatzin Ereth Robles-Chávez, Nayeli Goreti Nieto-Velázquez, María Elizbeth Alvarez-Sánchez

**Affiliations:** 1 Laboratorio de Patogénesis Celular Humana y Veterinaria, Posgrado en Ciencias Genómicas, Universidad Autónoma de la Ciudad de México (UACM), San Lorenzo 290, Col. Del Valle, Mexico City 09790, Mexico; jo_puenter@hotmail.com (J.P.-R.);; 2 División de Investigación, Hospital Juárez de México, Instituto Politécnico Nacional 5160, Col. Magdalena de las Salinas, Mexico City 07760, Mexico; goretinieto@gmail.com; 3 Departamento de Biología Celular y Fisiología, Instituto de Investigaciones Biomédicas, Universidad Nacional Autónoma de México, Mexico City 04510, Mexico; iraiz.nunez@iibiomedicas.unam.mx

**Keywords:** prostate cancer, angiogenesis, miRNAs, lncRNAs, metastasis, transcriptomics

## Abstract

Prostate cancer (PCa) is a leading cause of cancer-related mortality in men and is often characterized by aggressive growth and bone metastasis. Angiogenesis plays a central role in tumor progression and dissemination. This study aimed to explore the regulatory roles of long non-coding RNAs (lncRNAs) and microRNAs (miRNAs) in angiogenesis and metastasis during PCa progression. Publicly available RNA-seq datasets were analyzed to identify differentially expressed miRNAs between metastatic (N1) and nonmetastatic (N0) PCa. Bioinformatic tools were used to reconstruct co-regulatory networks involving miRNAs, lncRNAs, and angiogenesis-related mRNAs. RT-qPCR was performed on serum-derived liquid biopsies from N0 and N1 patients and healthy controls to validate the key regulatory axes. Transcriptomic analysis revealed that miRNAs such as hsa-miR-183-5p and hsa-miR-216a-5p were upregulated in N1 PCa and associated with pro-angiogenic signaling, whereas hsa-miR-206 and hsa-miR-184, known for their anti-angiogenic functions, were downregulated. Network analysis identified the LINC00261–miR-206–HIF1A axis as the central regulatory module. RT-qPCR validation confirmed the significant downregulation of LINC00261 and miR-206, along with HIF1A overexpression in N1 samples compared to N0 and controls (*p* < 0.001), supporting in silico predictions. These findings highlight the role of ncRNA-mediated regulation of PCa angiogenesis and metastasis. The LINC00261–miR-206–HIF1A axis may serve as a promising noninvasive biomarker and potential therapeutic target. The integration of computational and experimental data provides a strong rationale for the further functional validation of advanced PCa.

## 1. Introduction

Prostate cancer (PCa) is a multifactorial malignancy and a leading cause of cancer-related death among men worldwide [1]. While some tumors remain indolent, others progress to aggressive metastatic forms, frequently spreading to bone, and are associated with poor prognosis and therapeutic resistance [2,3]. Metastasis involves epithelial-to-mesenchymal transition (EMT), extracellular matrix remodeling, and vascular dissemination. Angiogenesis plays a central role in supporting tumor growth and dissemination [4,5]. Angiogenesis is driven by a balance between pro- and anti-angiogenic factors, a balance often disrupted in cancer [6,7]. Vascular endothelial growth factor (VEGF) is a key pro-angiogenic molecule, and its inhibition has shown clinical benefits; however, tumors commonly develop resistance through alternative pathways and adaptation to hypoxia [8,9,10,11]. The heterogeneous nature of PCa at the molecular and clinical levels complicates treatment responses, particularly during androgen deprivation therapy (ADT) [12,13,14].

Long non-coding RNAs (lncRNAs) and microRNAs (miRNAs), two major classes of non-coding RNAs, have emerged as critical modulators of angiogenic signaling and tumor progression [13,14,15]. LncRNAs can function as competitive endogenous RNAs (ceRNAs), sequestering miRNAs and thus regulating the availability of miRNAs to target angiogenesis-related mRNAs [16,17]. In PCa, aberrant expression of several miRNAs and lncRNAs has been associated with poor prognosis, metastasis, and resistance to therapy [18,19,20]. However, their integrated contribution to angiogenesis and nodal metastasis has not yet been sufficiently explored.

Recent studies have highlighted the regulatory functions of non-coding RNAs—specifically long non-coding RNAs (lncRNAs) and microRNAs (miRNAs)—in PCa progression, angiogenesis, and metastasis [15,16]. miRNAs (20–24 nucleotides) regulate gene expression by targeting 3′UTRs, whereas lncRNAs (>200 nucleotides) function via diverse epigenetic and post-transcriptional mechanisms [17,18]. These lncRNAs often function as competing endogenous RNAs (ceRNAs) or “sponges” that sequester tumor-suppressive miRNAs and facilitate oncogenic signaling. Clinically, dysregulated lncRNA–miRNA axes have been implicated in progression toward more aggressive and treatment-resistant phenotypes. For example, lncRNAs such as HOTAIR and miR-34a are involved in the development of resistance to androgen deprivation therapy by modulating the cell cycle and angiogenesis pathways [19].

These RNAs interact in complex networks, often with lncRNAs acting as competing endogenous RNAs (ceRNAs) that sequester miRNAs and modulate mRNA translation [4,20]. Depending on the cellular context and target genes, miRNAs can function as either oncogenes or tumor suppressors [21,22,23,24,25]. Despite significant advances, comprehensive characterization of lncRNA–miRNA–mRNA regulatory circuits in PCa remains limited. Many studies have focused on isolated interactions or in vitro models, leaving gaps in our understanding of how these networks orchestrate the angiogenic and metastatic phenotypes in vivo. Furthermore, tumor microenvironmental conditions such as hypoxia and oxidative stress appear to modulate these regulatory pathways, adding additional complexity. Growing evidence suggests that miRNAs are integral regulators of angiogenesis and metastatic progression in PCa [26,27,28,29]. Comprehensive profiling of lncRNA–miRNA–mRNA regulatory circuits in the context of the tumor microenvironment, particularly under hypoxia or oxidative stress, remains limited, many of which are involved in modulating angiogenic signaling. For example, the expression of miR-221 and miR-222 in endothelial cells has been shown to influence angiogenic activity by targeting receptors like c-Kit [20,30,31], thereby modulating responses to angiogenic ligands such as stem cell factor (SCF). Subsequent profiling studies identified over 200 miRNAs expressed in the vascular endothelium, with a core set of approximately 28 miRNAs consistently reported across multiple datasets [32].

Therefore, the objective of this study was to systematically characterize angiogenesis-associated non-coding RNA regulation in metastatic versus non-metastatic PCa by integrating publicly available RNA-seq datasets. Specifically, we (i) identified differentially expressed angiogenesis-related miRNAs associated with nodal metastasis status, (ii) reconstructed lncRNA–miRNA–mRNA co-regulatory networks using curated and predictive interaction resources, and (iii) performed a preliminary RT-qPCR assessment of selected axis components in serum-derived liquid biopsy samples from patients with PCa.

## 2. Materials and Methods

### 2.1. Public RNA-seq Data of miRNAs and mRNAs

RNA-seq data for miRNAs from 19 metastatic and 19 non-metastatic PCa samples (GSE117674) were downloaded from the Gene Expression Omnibus (GEO) database. Differential expression analysis was performed using DESeq2 based on negative binomial distribution. miRNAs were considered differentially expressed with a fold change ≥ 1.5 and a *p*-value < 0.05. Subsequently, the target mRNAs of the differentially expressed miRNAs involved in the negative regulation of angiogenesis (GO: 0016525) and positive regulation of angiogenesis (GO:0045766) were retrieved from the ENCORI database (https://rnasysu.com/encori/) (accessed on 10 May 2025) [33], which integrates predictive data from resources such as PicTar and TargetScan. We identified five upregulated miRNAs in metastatic patients that target mRNAs associated with the negative regulation of angiogenesis, and five downregulated miRNAs targeting mRNAs involved in the positive regulation of angiogenesis. The regulatory network between metastasis-associated miRNAs and angiogenesis-related genes was visualized using Cytoscape v3.10.4 (https://cytoscape.org/) (accessed on 16 June 2025).

### 2.2. TCGA-PRAD Data Analysis

TCGA-PRAD data, including clinical stratification for N0 and N1 stages and miRNA/mRNA expression profiles, were downloaded from the UCSC Xena platform (http://xena.ucsc.edu/) (accesed on 13 May 2025). The miRNAs differentially expressed between the N0 and N1 groups were selected for further analysis. Pathway enrichment analysis was performed using the MSigDB Hallmark 2020 collection through the Enrichr database, based on the predicted mRNA targets of two miRNAs upregulated in metastasis (miR-183-5p and miR-216a-5p) and two miRNAs downregulated in metastatic patients (miR-184 and miR-206).

### 2.3. Public RNA-seq Data of lncRNAs

RNA-seq data for lncRNA expression from an experimental model of angiogenesis inhibition in CRPC C4-2 cells (GSE172205) were downloaded. Differential expression of lncRNAs was assessed using DESeq2 with a significance threshold of fold change ≥ 1.5 and *p*-value < 0.05, compared to controls.

### 2.4. Bioinformatic Analysis

Bioinformatic analyses included the generation of volcano plots and hierarchical clustering heatmaps. To further explore regulatory interactions, miRNA-lncRNA interaction analysis was conducted using the miRNet database (https://www.mirnet.ca/) (accessed on 25 June 2025), which compiles experimentally validated interactions from the CLIP-Seq and Degradome-Seq datasets. We investigated the interactions between miR-183-5p, miR-216a-5p, miR-184, and miR-206. The resulting regulatory network was visualized using Cytoscape software.

### 2.5. Liquid Biopsy Collection and RNA Extraction

Liquid biopsies were obtained from male patients with prostate cancer (PCa), classified as N0 (*n* = 6) or N1 (*n* = 6), and from healthy controls (*n* = 6) recruited at Hospital Juárez de México under the approved protocol HJM-009/23-I. After obtaining informed consent, 4–6 mL of peripheral blood was collected in BD Vacutainer^®^ serum tubes (Becton, Dickinson and Company, Franklin Lakes, NJ, USA), centrifuged at 300× *g* for 5 min at room temperature to isolate serum, and stored at −80 °C for 24 h. The inclusion criteria for PCa cases included men over 40 years of age, PSA > 4 ng/mL, and Gleason score ≥ 6. Controls were healthy men aged > 40 years with PSA < 4 ng/mL and no family history of PCa or catastrophic illness. Hemolyzed samples and individuals with other malignancies were excluded from this study.

Total RNA was extracted from 200 µL of serum using TRIzol™ reagent (Invitrogen™, Thermo Fisher Scientific, Waltham, MA, USA). Briefly, 1 mL TRIzol was added to the sample, mixed thoroughly, and transferred to a 1.5 mL microcentrifuge tube. After 5 min of incubation at room temperature, 200 µL of chloroform (Sigma-Aldrich, St. Louis, MO, USA) was added, followed by vigorous shaking for 15 s and centrifugation at 12,500 rpm for 25 min at 4 °C. The aqueous phase was transferred to a fresh tube, and RNA was precipitated with 500 µL isopropanol (J.T. Baker, Phillipsburg, NJ, USA), incubated on ice for 20 min, and centrifuged again under the same conditions. The resulting pellet was washed with 1 mL 75% ethanol (Merck, Darmstadt, Germany), air-dried for 5 min, and resuspended in 20 µL nuclease-free water (Ambion™, Thermo Fisher Scientific, Waltham, MA, USA). RNA concentration and purity were assessed by spectrophotometry using a NanoDrop 2000 (Thermo Fisher Scientific, Waltham, MA, USA), and RNA was used immediately for cDNA synthesis.

### 2.6. cDNA Synthesis and Quantitative Real-Time PCR (qRT-PCR)

Complementary DNA (cDNA) was synthesized from the total RNA extracted from liquid biopsies using the TaqMan™ MicroRNA Reverse Transcription Kit (Applied Biosystems™, Foster City, CA, USA), following the manufacturer’s instructions. The cDNA was stored at −80 °C until use. Quantitative real-time PCR (qRT-PCR) was performed using SYBR™ Green PCR Master Mix (Applied Biosystems™, Foster City, CA, USA) and specific primers (see Supplementary Table S1) on an Applied Biosystems™ 7500 Real-Time PCR System. All reactions were conducted in duplicate and included three technical replicates per sample. Small nuclear RNA RNU6 was used as an endogenous control for the normalization of miRNA and lncRNA expression.

### 2.7. Statistical Analysis

Differentially expressed miRNAs (DEMs) were identified using the limma package in the R statistical environment (version 4.2.2), with a fold-change threshold ≥ 1.5 and a *p*-value < 0.05. Differences in the expression of lncRNA–miRNA–target gene axes in liquid biopsies among the control, N0, and N1 groups were assessed using one-way ANOVA followed by Tukey’s post hoc test and unpaired two-tailed *t*-tests, with *p* < 0.001 as statistically significant. Relative expression was calculated using the 2^−ΔΔCt^ method, normalized to RNU6, and reported as fold-change. All statistical analyses and graphs were generated using GraphPad Prism (version 9.3.0; GraphPad Software, San Diego, CA, USA). Statistically significant values are denoted by asterisks in the figures, with the corresponding *p*-values detailed in the figure legends.

## 3. Results

### 3.1. Differentially Expressed miRNAs Regulating Angiogenesis in Metastatic Prostate Cancer

**Figure 1:**
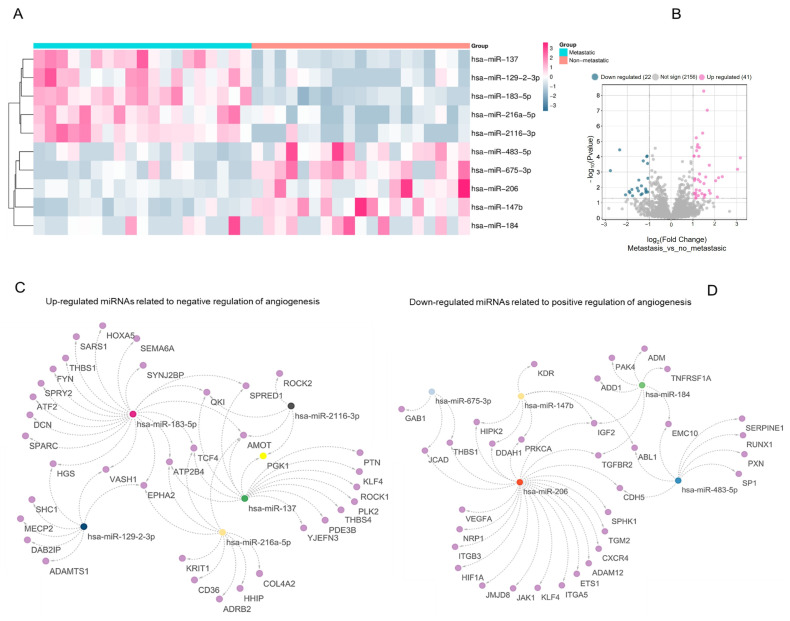
Transcriptomic profile and interaction networks of angiogenesis-related miRNAs in metastatic vs. non-metastatic PCa (**A**) Heatmap of differentially expressed miRNAs in metastatic and non-metastatic samples from the GSE117674 dataset. Color intensity reflects normalized z-scores, with red indicating high relative expression and blue indicating low expression. Highlighted miRNAs are significantly deregulated (*p* < 0.05; |log_2_FC| > 1) and functionally associated with angiogenesis. (**B**) Volcano plot of miRNA expression differences between metastatic and non-metastatic samples. The *X*-axis denotes log_2_ fold change; the *Y*-axis indicates –log_10_ *p*-values. Statistically significant upregulated (pink) and downregulated (blue) miRNAs are emphasized. (**C**) Interaction network of upregulated miRNAs targeting genes involved in the negative regulation of angiogenesis, illustrating the interaction network toward a pro-angiogenic phenotype. Nodes represent miRNAs (triangles) and predicted/validated target genes (circles); edges denote interaction evidence. (**D**) Interaction network of downregulated miRNAs targeting genes associated with the positive regulation of angiogenesis showing reduced expression of these miRNAs in metastatic progression.

To explore the regulatory involvement of miRNAs in the angiogenic switch during PCa progression, we compared miRNA expression profiles between metastatic and non-metastatic samples, focusing on those targeting genes annotated under Gene Ontology (GO) terms related to angiogenesis regulation. Among the upregulated miRNAs, hsa-miR-137, hsa-miR-129-2-3p, hsa-miR-183-5p, hsa-miR-216a-5p, and hsa-miR-2116-3p were predicted to be target genes associated with the “negative regulation of angiogenesis” (Figure 1A–C and Figure S1). The analysis also revealed 63 differentially expressed miRNAs in metastatic PCa: 41 were upregulated and 22 were downregulated (fold-change ≥ 1.5, *p* < 0.05) (Figure 1B). Conversely, hsa-miR-483-5p, hsa-miR-675-3p, hsa-miR-206, hsa-miR-147b, and hsa-miR-184 were downregulated and linked to genes annotated under ‘positive regulation of angiogenesis’ (Figure 1D; Supplementary Table S1). Within this interaction network, hsa-miR-206 displayed extensive predicted connectivity with pro-angiogenic targets (Figure 1D).

### 3.2. Expression and Functional Profiling of Upregulated miRNAs Targeting Negative Regulators of Angiogenesis

**Figure 2:**
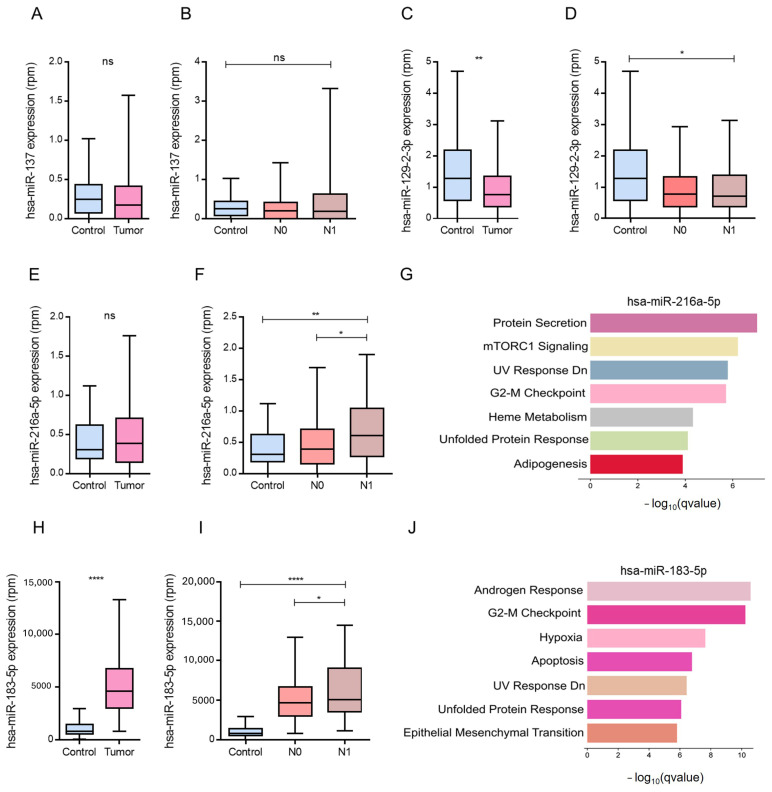
Expression patterns of miRNAs involved in the negative regulation of angiogenesis and pathway enrichment in PCa metastasis. (**A**–**F**,**H**,**I**) Boxplots represent normalized expression levels (ppm, parts per million) for selected miRNAs in control versus tumor tissues (**A**,**C**,**E**,**H**) and across lymph node metastasis stages (N0 = non-metastatic; N1 = metastatic) (**B**,**D**,**F**,**I**). Statistical significance is indicated as follows: * *p* < 0.05, ** *p* < 0.01, **** *p* < 0.0001; ns = not significant. (**G**,**J**) Hallmark pathway enrichment analysis based on predicted targets of miR-216a-5p (**G**) and miR-183-5p (**J**). Enrichment in non-metastatic (N0) samples included protein secretion, mTORC1 signaling, and the unfolded protein response (**G**), while androgen response, G2-M checkpoint, hypoxia, and epithelial–mesenchymal transition were prominent in metastatic (N1) samples (**J**).

Using TCGA-PRAD expression and clinical data, we evaluated the levels of four miRNAs previously predicted to inhibit antiangiogenic genes: hsa-miR-137, hsa-miR-129-2-3p, hsa-miR-183-5p, and hsa-miR-216a-5p. Expression was compared between normal and tumor samples, as well as across nodal metastasis stages (non-metastatic, N0 vs. metastatic, N1). hsa-miR-137 did not show significant expression differences between tumor and control samples or between N0 and N1 stages (Figure 2A,B). In contrast, hsa-miR-129-2-3p was significantly downregulated in tumor samples compared to normal tissues (*p* < 0.05), but its expression did not differ between the N0 and N1 stages (Figure 2C,D). hsa-miR-216a-5p and hsa-miR-183-5p were both upregulated in metastatic samples and exhibited significantly higher expression in the N1 stage than in the N0 stage (Figure 2F,I). Interestingly, hsa-miR-216a-5p was not significantly upregulated in the tumor versus control samples (Figure 2E). In contrast, hsa-miR-183-5p showed consistent upregulation across all comparisons, including metastatic versus non-metastatic, tumor versus control (Figure 2H), and N1 versus N0 (Figure 2I).

To explore the biological functions of these miRNAs, we conducted pathway enrichment analysis using the MSigDB Hallmark database. The targets of hsa-miR-216a-5p were enriched in pathways related to protein secretion, mTORC1 signaling, and heme metabolism (Figure 2G). In parallel, hsa-miR-183-5p targets were significantly enriched in hallmark gene sets associated with the androgen response, previously linked to angiogenesis in prostate tumors [34,35]. Other important enriched terms for this miRNA included G2-M checkpoint, hypoxia, and epithelial–mesenchymal transition (Figure 2J).

### 3.3. Downregulated miRNAs Associated with Positive Regulation of Angiogenesis in Metastatic PCa

**Figure 3:**
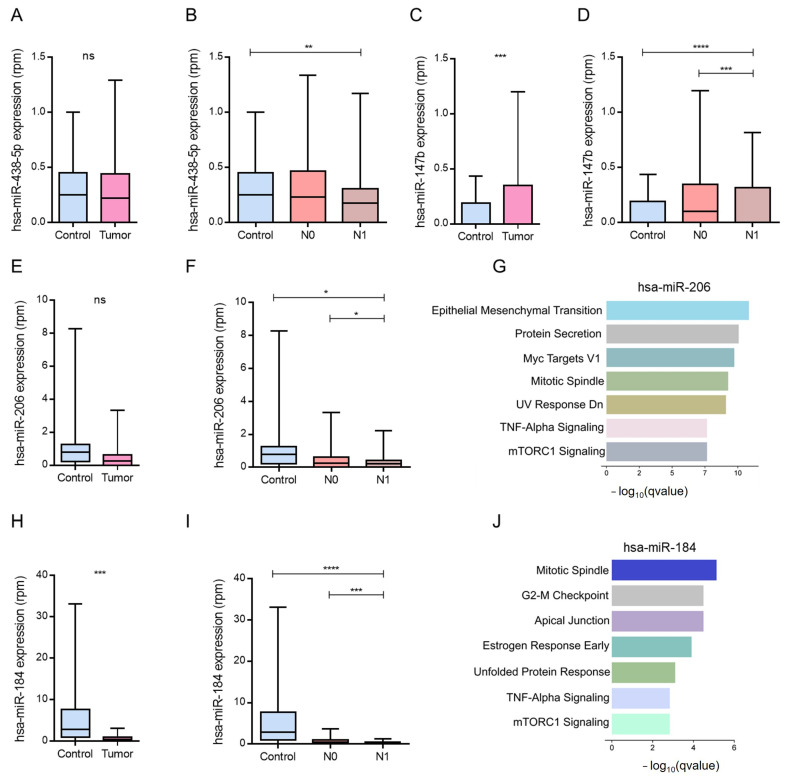
Differential expression of angiogenesis-related miRNAs and enrichment of pathways associated with tumor progression and metastasis in PCa. (**A**–**F**,**H**,**I**) Boxplots show normalized miRNA expression levels (ppm) in tumor versus control samples and stratified by metastatic status (N0 = non-metastatic; N1 = metastatic). Statistical significance is indicated by asterisks: * *p* < 0.05, ** *p* < 0.01, *** *p* < 0.001, **** *p* < 0.0001; ns = not significant. (**G**) Enrichment analysis in metastatic samples highlights pathways including epithelial–mesenchymal transition (EMT), protein secretion, and TNF-α signaling. (**J**) Pathways enriched in tumor versus normal tissue include adipogenesis, E2F targets, androgen response, and G2-M checkpoint. Enriched terms in advanced metastasis emphasize mitotic spindle formation, apical junction remodeling, estrogen signaling, and unfolded protein response.

Next, we examined the expression patterns of miRNAs downregulated in metastatic PCa, which are known to be involved in the positive regulation of angiogenesis. Using the TCGA-PRAD data, we focused on hsa-miR-483-5p, hsa-miR-147b, hsa-miR-206, and hsa-miR-184. Among these, hsa-miR-483-5p showed no significant differences in expression between tumor and control samples or between the N0 and N1 stages (Figure 3A,B). Although hsa-miR-147b was repressed in metastatic profiles, its expression was paradoxically elevated in tumor tissues compared to normal controls, with no differences between N0 and N1 stages (Figure 3C,D).

In contrast, hsa-miR-184 was significantly downregulated in both tumor samples and metastatic (N1) stages, which is consistent with its previously described tumor-suppressive role (Figure 3H,I). Similarly, hsa-miR-206, which was reduced in metastatic samples, showed significantly lower expression in N1 than in N0 (Figure 3F).

To explore their functional relevance, we conducted enrichment analysis of the predicted target genes. For hsa-miR-206, results highlighted the key pathways involved in epithelial–mesenchymal transition (EMT), MYC signaling, protein secretion, estrogen response, and apical junction organization (Figure 3G–J).

### 3.4. Modulation of Negative Angiogenesis-Related Genes by hsa-miR-183-5p and hsa-miR-216a-5p in PCa

**Figure 4:**
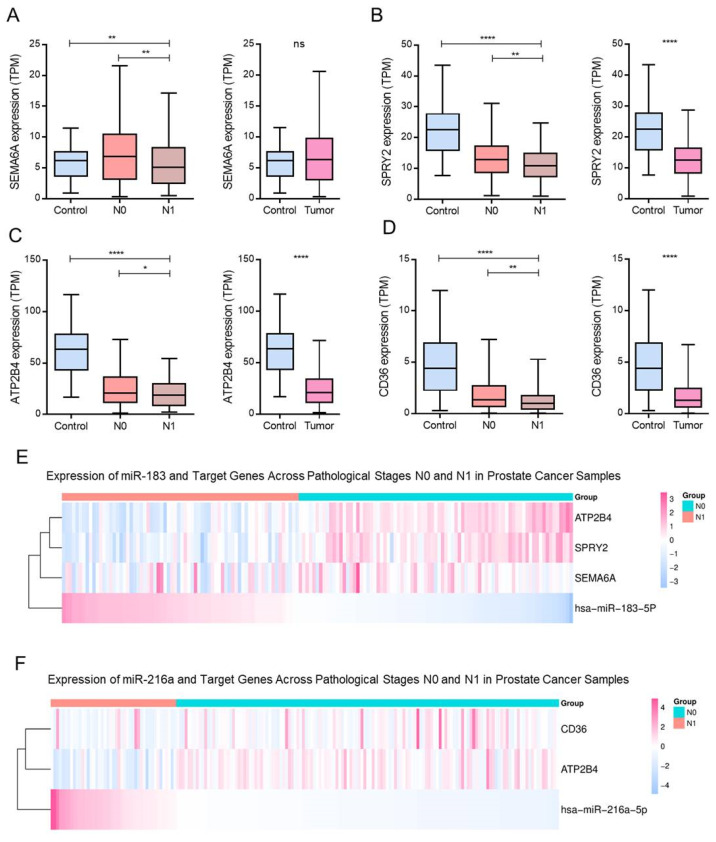
Expression profiles of hsa-miR-183-5p, hsa-miR-216a-5p, and their predicted anti-angiogenic target genes in PCa samples across clinical stages. (**A**–**C**,**E**,**F**) Boxplots display normalized transcript levels (TPM: transcripts per million) of *SEMA6A*, *SPRY2*, *ATP2B4*, and *CD36* in control, N0 (non-metastatic), and N1 (metastatic) samples, as well as tumor versus control comparisons. Statistical significance is indicated as follows: * *p* < 0.05, ** *p* < 0.01, **** *p* < 0.0001; ns = not significant. (**D**) Heatmap illustrating expression profiles of hsa-miR-183-5p and its associated targets (*SEMA6A*, *FYN*, *SPRY2*, and *ATP2B4*) across control, N0, and N1 samples. Values are z-score-normalized. (**F**) Heatmap showing hsa-miR-216a-5p and its predicted targets (*ATP2B4*, *CD36*) across the same sample groups. Z-score normalization highlights relative expression shifts by pathological stage.

We analyzed genes involved in the negative regulation of angiogenesis that were significantly downregulated in the N1-stage samples of PCa from TCGA-PRAD. Among these, *SEMA6A*, *SPRY2*, *ATP2B4*, and *CD36* exhibited consistent reductions under metastatic conditions (Figure 4A–D). Notably, *SPRY2* and *ATP2B4* were more highly expressed in normal tissues than in tumor tissues (Figure 4B,C), while *CD36* showed decreased expression in PCa and further reduction in N1 compared to N0 (Figure 4D).

Although *SEMA6A* expression did not differ significantly between the tumor and normal tissues, its levels were markedly lower in N1 than in N0 (Figure 4A). These transcriptional shifts were inversely related to the miRNA expression patterns. Specifically, hsa-miR-183-5p showed increased expression in N1 samples, whereas its putative targets, *SPRY2* and *ATP2B4* were downregulated (Figure 4E). In contrast, in N0 samples, where hsa-miR-183-5p expression was lower, these angiogenesis-inhibiting genes were expressed at higher levels.

A similar inverse correlation was observed between hsa-miR-216a-5p and *ATP2B4* expression (Figure 4F) Overall, the comparisons showed opposing expression patterns between hsa-miR-183-5p/hsa-miR-216a-5p and the corresponding predicted anti-angiogenic target genes across N0 and N1 samples.

### 3.5. Modulation of Positive Angiogenesis-Related Genes by hsa-miR-206 and hsa-miR-184

**Figure 5:**
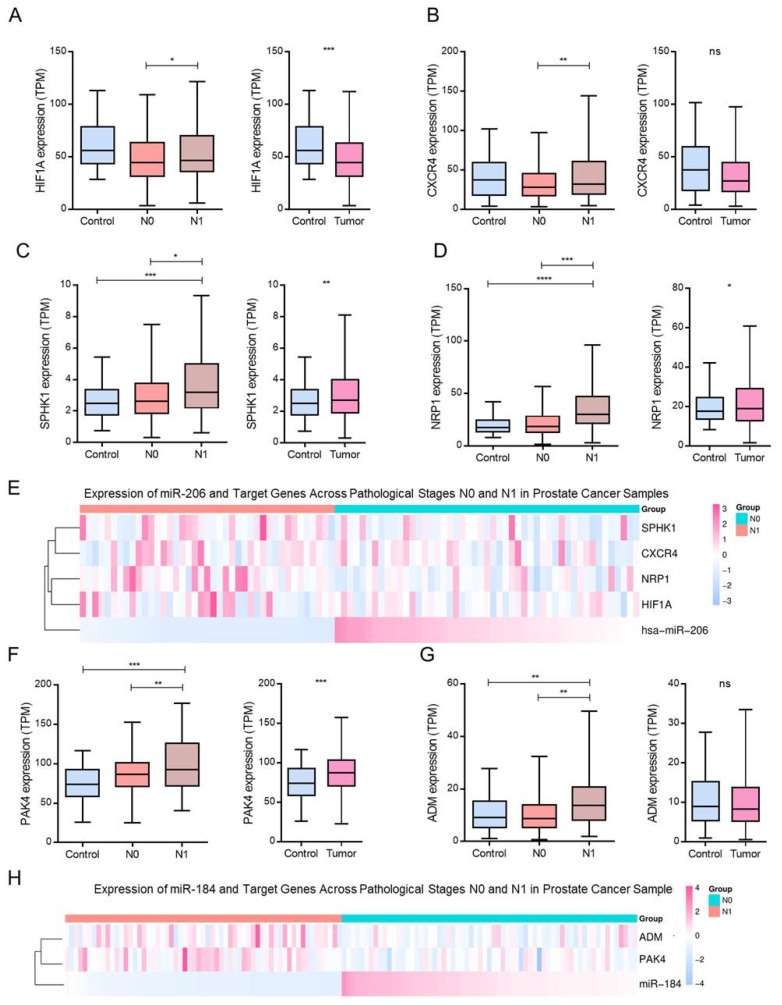
Expression analysis of miR-206 and miR-184 target genes in PCa samples across pathological stages. Boxplots display normalized expression levels (TPM: transcripts per million) of predicted or validated targets of hsa-miR-206 and hsa-miR-184 across control, non-metastatic (N0), and metastatic (N1) PCa samples. Statistical significance is denoted as: * *p* < 0.05, ** *p* < 0.01, *** *p* < 0.001, **** *p* < 0.0001; ns = not significant. (**A**) *HIF1A* expression in tumors and N1 samples vs. controls. (**B**) *CXCR4* expression in N0 vs. N1, and tumor vs. control comparisons. (**C**) *SPHK1* expression across pathological stages and control samples. (**D**) *NRP1* upregulation in metastatic (N1) samples relative to N0. (**E**) Heatmap showing expression patterns of hsa-miR-206 and its targets (*SPHK1*, *CXCR4*, *NRP1*, *HIF1A*) across pathological groups. Color intensity reflects z-score normalization. (**F**) *PAK4* expression: elevated in both N0 and N1 samples compared to controls and markedly increased in tumors. (**G**) *ADM* expression in N0 and N1 samples relative to controls. (**H**) Heatmap illustrating the expression of hsa-miR-184 and its targets (*PAK4*, *ADM*) across PCa groups. Data are shown as z-scores, emphasizing their progressive upregulation in metastatic conditions.

To explore the molecular basis underlying proangiogenic mechanisms in metastatic PCa, we analyzed the expression of genes annotated under the positive regulation of angiogenesis in TCGA-PRAD samples stratified by clinical stage. Several of these genes, *HIF1A*, *CXCR4*, *SPHK1*, *NRP1*, *PAK4*, and *ADM*, were identified as targets of the downregulated miRNAs hsa-miR-206 and hsa-miR-184, and exhibited increased expression in metastatic (N1) samples (Figure 5A,D,F,G). Specifically, *HIF1A* and *CXCR4* were significantly upregulated in N1 compared to N0 (Figure 5A,B), and similar trends were observed for *SPHK1* and *NRP1* (Figure 5C,D).

An inverse expression pattern was evident when hsa-miR-206 and its predicted mRNA targets were compared. In non-metastatic (N0) patients, where hsa-miR-206 is relatively overexpressed, the expression of *HIF1A*, *CXCR4*, *SPHK1*, and *NRP1* is reduced. In contrast, in metastatic (N1) samples, hsa-miR-206 levels were repressed, coinciding with the upregulation of its pro-angiogenic targets (Figure 5E).

A similar pattern was noted for hsa-miR-184 and its associated targets, *PAK4* and *ADM*, which were significantly upregulated in N1 samples compared to N0 samples. This inverse correlation supports a model in which downregulation of hsa-miR-184 contributes to derepression of angiogenic genes during metastasis (Figure 5H). Together, these analyses show lower expression of hsa-miR-206 and hsa-miR-184 in N1 samples, alongside higher expression of several predicted target genes within the ‘positive regulation of angiogenesis’ category, including *HIF1A*, *CXCR4*, *SPHK1*, *NRP1*, *PAK4*, and *ADM* (Figure 5).

### 3.6. Co-Regulation Network Between lncRNAs and miRNAs Associated with the Positive and Negative Regulation of Angiogenesis

To better characterize the regulatory networks involved in angiogenesis and metastatic progression in PCa, we conducted an integrative network analysis of potential lncRNA–miRNA–mRNA interactions. Using the MiRNet platform, we identified lncRNAs predicted to interact with key angiogenesis-associated miRNAs: hsa-miR-183-5p, hsa-miR-216a-5p, hsa-miR-184, and hsa-miR-206.

We first examined miRNAs upregulated in metastatic PCa, particularly hsa-miR-183-5p and hsa-miR-216a-5p, which are linked to the repression of anti-angiogenic genes, such as *SEMA6A*, *SPRY2*, *ATP2B4*, and *CD36*. These miRNAs were connected to 66 predicted lncRNAs (Figure S1A), with hsa-miR-216a-5p showing a notably broader interaction profile than hsa-miR-183-5p.

In parallel, we analyzed miRNAs downregulated in metastatic samples, hsa-miR-206 and hsa-miR-184, which are known to target genes associated with the promotion of angiogenesis, including *ADM*, *PAK4*, *SPHK1*, *CXCR4*, *HIF1A*, and *NRP1*. These miRNAs were linked to 52 predicted lncRNAs (Figure S1B), highlighting the complexity of post-transcriptional regulation in metastatic diseases. Notably, hsa-miR-206 exhibited the highest number of predicted lncRNA interactions among all miRNAs evaluated. Using MiRNet, we identified lncRNAs predicted to interact with hsa-miR-183-5p, hsa-miR-216a-5p, hsa-miR-184, and hsa-miR-206. hsa-miR-216a-5p and hsa-miR-183-5p were connected to 66 predicted lncRNAs (Figure S1A). In parallel, hsa-miR-206 and hsa-miR-184 were linked to 52 predicted lncRNAs (Figure S1B).

### 3.7. Integration of lncRNA Expression Profiles and Regulatory Networks in Angiogenesis Suppression

**Figure 6:**
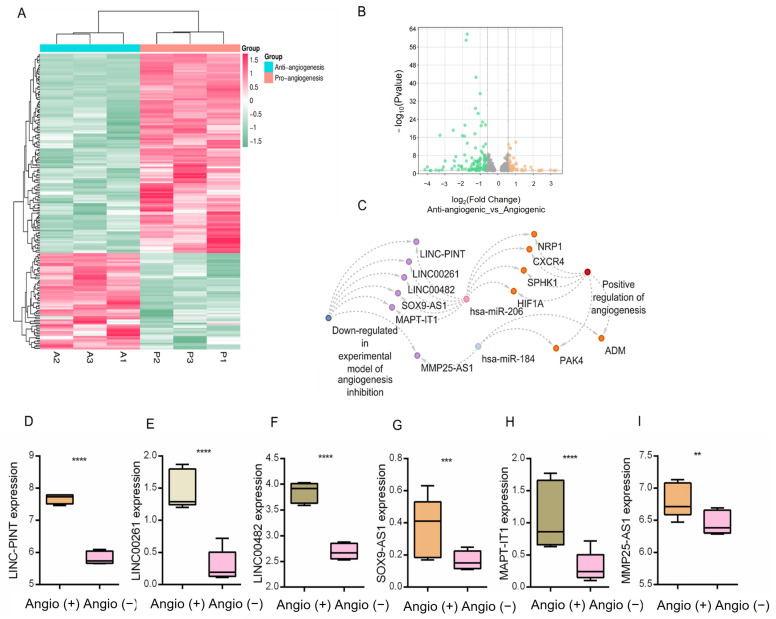
Co-regulation network between lncRNAs and miRNAs associated with angiogenesis under angiogenesis-inhibited conditions. (**A**) Heatmap showing the expression profiles of lncRNAs in the angiogenesis inhibition model (GSE172205). Expression values are scaled as z-scores to visualize up- and down-regulated transcripts. (**B**) Volcano plot of differentially expressed lncRNAs, highlighting upregulated (orange) and downregulated (green) transcripts with statistical significance (*p* < 0.05) The *x*-axis represents Fold Change, and the *y*-axis represents −log_10_ (*p*-value). Vertical dashed lines indicate the fold-change thresholds, and the horizontal dashed line indicates the statistical significance cutoff. Points are colored according to their differential expression status. (**C**) Regulatory network displaying the interactions between hsa-miR-206 and hsa-miR-184 and their associated lncRNAs under angiogenesis-inhibited conditions. Colors indicate the direction of regulation: down-regulated molecules in the anti-angiogenic condition are shown in purple dots, whereas up-regulated pro-angiogenic-associated molecules are shown in orange dots. Dashed edges represent predicted or reported regulatory interactions. (**D**–**I**) Boxplots of lncRNA expression levels (LINC-PINT, LINC00261, LINC00482, SOX9-AS1, MAPT-IT1, and MMP25-AS1) comparing angiogenesis-inhibited [Angio (–)] and angiogenesis-active [Angio (+)] states. Asterisks indicate statistical significance: ** *p* < 0.01, *** *p* < 0.001, **** *p* < 0.0001.

To explore the functional relevance of lncRNAs in the suppression of angiogenesis, we analyzed RNA-seq data from the publicly available GSE172205 dataset, which models the conditions of angiogenesis inhibition. Differential expression analysis was performed using the DESeq2 package, applying a fold change cutoff of ≥1.5 and a *p*-value < 0.05. This analysis identified 120 downregulated and 58 upregulated lncRNAs under antiangiogenic conditions (Figure 6A,B).

We then integrated these differentially expressed lncRNAs with the predicted lncRNA–miRNA interactions identified using MiRNet, focusing on angiogenesis-related miRNAs. This allowed for the construction of two regulatory interaction networks. One network centered on lncRNAs that were suppressed in the angiogenesis inhibition model included *MMP25-AS1*, *LINC-PINT*, *MAPT-IT1*, *SOX9-AS1*, *LINC00482*, and *LINC00261* (Supplementary Figure S2). These lncRNAs were predicted to interact with hsa-miR-206 and hsa-miR-184, which have been previously shown to be upregulated in non-metastatic (N0 stage) PCa samples.

In this network, higher miR-206 and miR-184 levels in N0 samples were accompanied by lower expression of several pro-angiogenic genes, including *SPHK1*, *NRP1*, *HIF1A*, *CXCR4*, *PAK4*, and *ADM* (Figure 6C).

### 3.8. Validation of the LINC00261–miR-206–HIF1A Axis in Liquid Biopsy Samples by RT-qPCR

Among the multiple regulatory circuits identified through bioinformatic analyses, the LINC00261–miR-206–HIF1A axis has emerged as a particularly compelling candidate for preliminary experimental validation due to its coherent expression patterns, functional relevance to angiogenesis, and robust integrative evidence from both transcriptomic and network-based approaches.

First, LINC00261 was one of the most significantly downregulated lncRNAs in the angiogenesis inhibition model (GSE172205) and was consistently linked to miR-206, a microRNA that was found to be downregulated in metastatic PCa samples (N1) but upregulated in non-metastatic cases (N0). Moreover, HIF1A, a well-established angiogenic driver and validated target of miR-206, was concurrently upregulated in metastatic samples and was tightly associated with the angiogenic switch.

**Figure 7:**
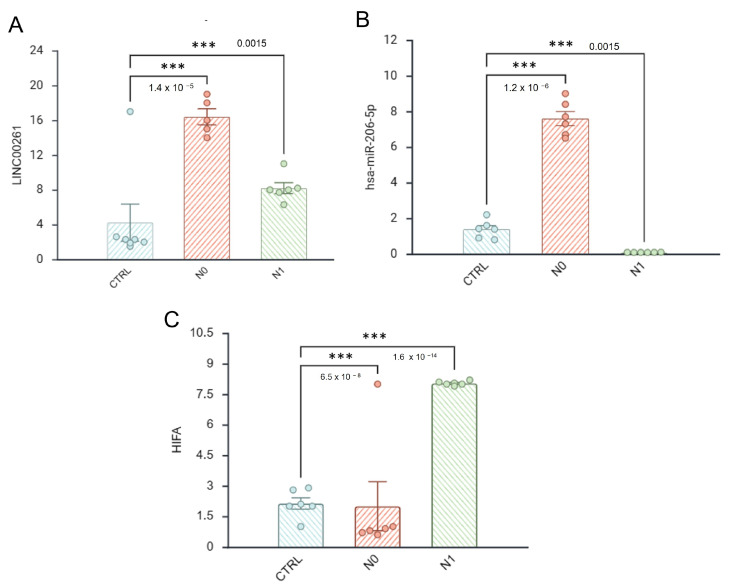
Relative expression of the LINC00261–miR-206–HIF1A axis in liquid biopsy samples from PCa patients. (**A**) *LINC00261*, (**B**) *hsa-miR-206*, and (**C**) *HIF1A* expression levels were measured in serum from healthy donors (CTRL), non-metastatic (N0), and metastatic (N1) PCa patients by RT-qPCR. Relative expression was calculated using the 2^−ΔΔCt^ method. Error bars represent ±standard deviation from two independent experiments performed in technical triplicates. Statistical significance was evaluated by one-way ANOVA. *** *p* < 0.0001. miRNA levels were normalized to snRNU6 expression.

To preliminarily assess the clinical detectability and stage-associated behavior of this regulatory module, we performed RT-qPCR on serum-derived liquid biopsy samples obtained from healthy donors (control, *n* = 5), patients with non-metastatic PCa (N0, *n* = 6), and patients with metastatic PCa (N1, *n* = 6). RNU6 was used as an internal control for normalization. Our results confirmed a significant downregulation of LINC00261 in metastatic (N1) samples compared to that in both N0 and control groups (ΔΔCt = +2.5 vs. N0; fold change = 0.177, *p* < 0.01), supporting its suppression during metastatic progression (Figure 7A). Interestingly, LINC00261 expression was higher in N0 than in controls. Consistent with the transcriptomic model, miR-206 was significantly repressed in N1 samples (ΔΔCt = +3.5 vs. N0; fold change = 0.088, *p* < 0.001) and moderately decreased in controls relative to N0 (fold-change = 0.4) (Figure 7B).

Conversely, HIF1A expression was strongly upregulated in N1 patients compared to N0 (ΔΔCt = −3.0; fold change = 8.0, *p* < 0.001), further supporting an inverse regulatory relationship with miR-206 (Figure 7C).

### 3.9. Integration of Upregulated lncRNA Expression and Regulatory Networks in Angiogenesis Suppression

**Figure 8:**
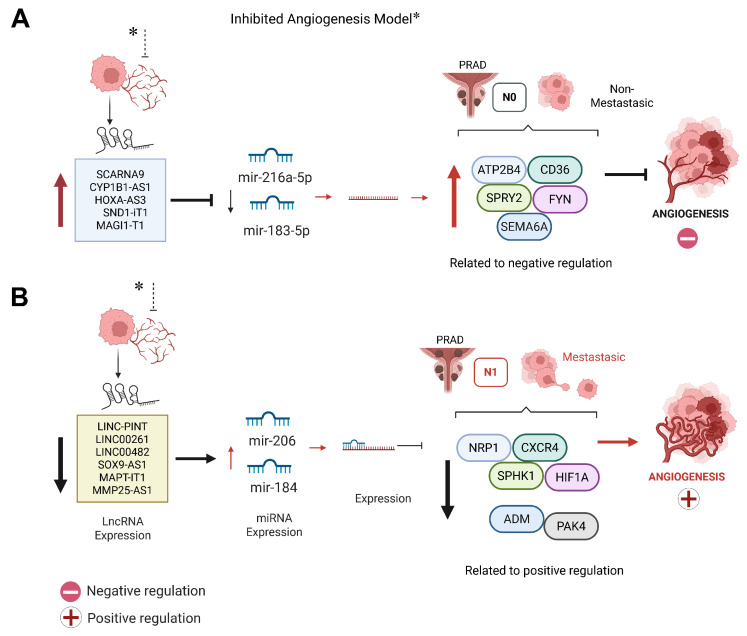
Integrated lncRNA–miRNA regulatory networks modulating angiogenesis in metastatic PCa. (**A**) In the context of angiogenesis inhibition and non-metastatic PCa (N0 stage), upregulated lncRNAs such as *SCARNA9*, *CYP1B1*-*AS1*, *HOXA-AS3*, *SND1-IT1*, and *MAGI1-IT1* are predicted to sequester miR-216a-5p and miR-183-5p. This suppression permits the upregulation of angiogenesis-inhibiting genes including *ATP2B4*, *CD36*, *SPRY2*, *FYN*, and *SEMA6A*, contributing to vascular suppression. (**B**) In contrast, metastatic PCa (N1 stage) is characterized by the downregulation of lncRNAs such as LINC-PINT, LINC00261, LINC00482, SOX9-AS1, MAPT-IT1, and MMP25-AS1, together with reduced miR-206 levels. RNA-seq analyses further suggest downregulation of miR-184 in N1. These changes are accompanied by increased expression of pro-angiogenic and pro-metastatic genes, including *NRP1*, *CXCR4*, *SPHK1*, *HIF1A*, *ADM*, and *PAK4*, consistent with a shift toward hypoxia-driven vascularization and metastatic progression. Red arrows denote positive regulation; black arrows indicate inhibitory interactions.

We integrated lncRNAs identified in the angiogenesis inhibition model with predicted lncRNA–miRNA interactions and angiogenesis-related mRNA targets to assemble an integrated regulatory network (Figure 8A and Figure S2). Specifically, *MAGI1-IT1*, *SND1-IT1*, *XIST*, *CYP1B1-AS1*, *HOXA-AS3*, and *SCARNA9* were predicted to interact with hsa-miR-183-5p and hsa-miR-216a-5p (Figure 8A). Figure 8B summarizes the RNA-seq-inferred expression profiles of these lncRNAs under angiogenesis-inhibited versus angiogenesis-promoting conditions.

### 3.10. Limitations of the Study and Future Directions

This study presents preliminary yet consistent findings on the regulatory landscape of non-coding RNAs in angiogenesis-associated programs linked to metastatic PCa/CRPC. Nevertheless, several limitations should be acknowledged. First, the analyses rely predominantly on in silico data from publicly available transcriptomic datasets, which may vary in clinical annotation, sample handling, and sequencing pipelines. Second, although a small cohort of serum-derived liquid biopsy samples from patients with PCa was included for RT-qPCR assessment of the proposed module, the limited sample size restricts generalizability and precludes definitive clinical conclusions.

In addition, all interaction networks were derived from predictive and curated databases without direct molecular confirmation. Therefore, the directionality and causality of the inferred relationships—particularly within the proposed LINC00261–miR-206–HIF1A module—cannot be established from the current dataset. The lack of functional assays (e.g., dual-luciferase reporter analyses and knockdown/overexpression or gain-/loss-of-function approaches) further limits mechanistic interpretation. Moreover, spatial and cell-type-specific expression was not assessed, which may critically influence angiogenic regulation in the tumor microenvironment.

Future studies will be required to mechanistically validate this module in PCa cell models, including LINC00261 perturbation using antisense oligonucleotides or siRNAs, and miR-206 gain- and loss-of-function experiments (mimics and anti-miRs). Following perturbation, LINC00261, miR-206, and HIF1A should be quantified at the transcript level (RT-qPCR) and HIF1A at the protein level, together with representative downstream hypoxia/angiogenesis targets (e.g., VEGFA). Direct targeting will also need to be tested using dual-luciferase reporter constructs containing the HIF1A 3′UTR with the predicted miR-206 binding site(s) and corresponding mutant controls. Finally, functional relevance should be assessed using phenotypic assays related to metastatic competence and angiogenesis (e.g., proliferation/viability, clonogenic growth, migration/invasion, and, when appropriate, endothelial tube-formation assays using conditioned media).

Despite these limitations, the present work provides testable hypotheses and highlights candidate regulatory axes with translational potential that merit further experimental and clinical evaluation.

## 4. Discussion

Overall, integrative network analyses highlighted the LINC00261–miR-206–HIF1A module as a candidate regulatory axis associated with nodal metastasis and pro-angiogenic signaling, supporting its prioritization for further mechanistic validation and evaluation in liquid biopsy settings. A central strength of this study lies in the selection of publicly available transcriptomic datasets, which provide a robust foundation for comparative and reproducible analyses. The integration of the GSE117674, GSE172205, and TCGA-PRAD datasets allowed the dissection of miRNA and lncRNA expression signatures in metastatic versus non-metastatic PCa, as well as in experimental models of angiogenesis inhibition. In parallel, ENCORI and miRNet platforms enabled the reconstruction of regulatory miRNA–lncRNA–mRNA networks with functional implications in angiogenesis.

Focusing on miRNA expression profiles, we observed that metastatic PCa was characterized by a transcriptional landscape enriched in angiogenesis-promoting signals. In particular, miR-183-5p and miR-216a-5p are overexpressed in metastatic samples, both of which have been associated with tumor aggressiveness in other cancers [36]. In breast cancer, miR-183-5p has been shown to enhance proliferation, metastasis, and angiogenesis by increasing VEGFA, N-cadherin, and vimentin expression while inhibiting p53 and E-cadherin expression through the negative regulation of FHL1, which subsequently reduces VEGF expression [37], while miR-216a-5p, although poorly described in PCa, acts as a tumor suppressor in breast and gastrointestinal cancers but has pro-angiogenic functions in cardiac tissue [38,39,40]. In contrast, miR-206 and miR-184, which are both downregulated in metastasis, have been implicated in anti-angiogenic regulation. miR-206 inhibits VEGFA expression and suppresses the migration of PCa and lung cancer cells [41,42]. Although no studies have directly associated miR-206 with angiogenesis in PCa, in lung cancer, this miRNA inhibits angiogenesis in NSCLC cells by blocking the 14-3-3ζ/STAT3/HIF-1α/VEGF signaling pathway, which is crucial for the formation of new blood vessels in tumors [43]. In contrast, miR-184 acts as a tumor suppressor in PCa by interfering with VEGFA induction via FOG2 repression, contributing to reduced neovascularization. Overexpression of miR-184 in DU145 cells also inhibited proliferation, migration, and invasion [44,45].

The interplay between miRNAs and lncRNAs adds another regulatory layer to this network. While several lncRNAs analyzed here lack direct experimental evidence in PCa, data from other cancers and pro-/anti-angiogenic models offer valuable insights. For instance, MMP25-AS1 and LINC-PINT showed expression patterns that mirrored angiogenic modulation in endothelial cells and increased the expression levels of VEGFC, MMP1, MMP24-AS1, and MMP25-AS1 in endothelial cells [46,47]. While SOX9-AS1 and LINC00482 were consistent with known roles in promoting vascular growth in breast and bladder cancer, respectively [48,49]. The inhibition of LINC00482 led to the negative regulation of VEGFA, resulting in the inhibition of angiogenesis [50]. Regarding the overexpressed lncRNAs in the angiogenesis inhibition model, the data obtained suggest that HOXA-AS3 acts as a negative regulator of angiogenesis because its overexpression is related to the inhibition of angiogenesis. These results coincide with previous findings observed in an in vitro model of atherosclerosis, where this lncRNA enhanced the ability of human vascular endothelial cells (HUVECs) to form blood vessel-like structures [51]. In parallel, we explored a second network of upregulated lncRNAs identified in the angiogenesis inhibition model, specifically *MAGI1-IT1*, *SND1-IT1*, *XIST*, *CYP1B1-AS1*, *HOXA-AS3*, and *SCARNA9* (Figure 8A and Figure S2). These lncRNAs were predicted to interact with hsa-miR-183-5p and hsa-miR-216a-5p, which displayed higher expression in N1 and comparatively lower levels in N0. Both miRNAs were linked, through curated/predictive resources, to angiogenesis-inhibiting genes such as *ATP2B4*, *SEMA6A*, *SPRY2*, and *CD36*. Collectively, this network provides a hypothesis-generating framework in which lncRNA–miRNA relationships may contribute to stage-associated differences in angiogenesis-related regulation.

Interestingly, our data indicated that an increase in XIST levels reduced angiogenesis. However, these results contradict the findings of previous studies. In models of hypoxia-induced angiogenesis in human brain microvascular endothelial cells (HBMEC), inhibition of XIST results in a decrease in angiogenesis [52], despite prior studies indicating that it promotes angiogenesis under hypoxic conditions or in glioma cells [53]. This discrepancy between our data and those reported in the literature may be due to the differences in the experimental models used. While we worked with an angiogenesis inhibition model, other studies have focused on the stimulation of angiogenesis under specific conditions, (such as hypoxia), which may influence the response profile of XIST. This discrepancy may arise from differences in cell types or experimental conditions, emphasizing the context-dependent behavior of this lncRNA. Although our results indicate an inhibitory role of XIST in angiogenesis, further studies are needed to clarify the specific biological context in which it operates. Likewise, while our data suggest that other lncRNAMAGI1-IT1, SND1-IT1, and CYP1B1-AS1 may be involved in regulating angiogenesis and metastasis in PCa, their roles remain unclear as no direct evidence exists in the current literature. Therefore, these findings should be interpreted with caution until validated. The expression profiles of each lncRNA under angiogenesis-inhibited versus angiogenesis-promoting conditions are shown in Figure 8B. Overall, the assembled co-regulatory networks suggest a modulatory contribution of these lncRNAs to the angiogenic balance during PCa progression; thus, they represent potential candidate biomarkers and therapeutic entry points. However, these relationships remain inferred and should be considered hypothesis-generating until validated through mechanistic assays and independent clinical cohorts. The emerging relevance of miRNAs, such as miR-107 and miR-182, further underscores their complex regulatory environment. miR-107, which is downregulated in metastatic PCa, acts as a tumor suppressor by targeting HIF-1α, thereby impairing pro-angiogenic signaling [54]. Conversely, miR-182 promotes angiogenesis through the suppression of PHD2 and FIH1, thereby enhancing HIF-1α and VEGF activity [55]. These findings highlight the dual roles that miRNAs can exert, depending on target specificity and microenvironmental context [56]. Emerging technologies, particularly those based on CRISPR-Cas9 functional screening, have revealed novel miRNA-lncRNA axes that modulate metastasis and resistance in cancer [57,58,59]. Additionally, the role of tumor-associated microbiota in regulating miRNA expression and therapy resistance in CRPC is gaining attention [60].

In our proposed model, early-stage PCa (N0) retains a relatively anti-angiogenic expression profile, characterized by higher levels of miRNAs such as miR-206, miR-1-3p, miR-143-3p, and miR-145-5p, together with inferred lncRNA-centered regulatory interactions involving LINC00261, LINC00665, and TUG1 that may influence miRNA–target relationships within the network [42,45,50]. Because these interactions are inferred from expression data and curated/predictive resources, we refer to this as a putative regulatory landscape that warrants mechanistic validation. This landscape shifts markedly in metastatic disease (N1), where pro-angiogenic genes such as *HIF1A*, *CXCR4*, and *VEGFA* are upregulated, along with a broader attenuation of regulatory non-coding RNAs [41,51,52].

Within this context, the LINC00261–miR-206–HIF1A axis has emerged as a key module linking non-coding RNA regulation to pro-angiogenic signaling. Preliminary RT-qPCR validation in serum-derived liquid biopsies supported this model, revealing strong repression of LINC00261 and miR-206 in N1 samples and the concurrent overexpression of HIF1A. Taken together, these data support the presence of a stage-associated LINC00261–miR-206–HIF1A expression pattern in serum-derived liquid biopsies. While the inverse relationship between miR-206 and HIF1A is consistent with miRNA-mediated regulation, the concurrent repression of LINC00261 and miR-206 in N1 does not allow inferring a ceRNA “sponge” mechanism or directionality from expression data alone. Therefore, we refer to this axis as a putative regulatory module that warrants mechanistic validation in PCa models. Notably, the selection of this axis was not solely based on differential expressions but also on its high node degree and centrality within the inferred co-regulatory network, reinforcing its relevance as a central regulatory hub in the transcriptional and post-transcriptional control of angiogenesis. Interestingly, miR-206 levels showed a stage-associated decrease, with the lowest levels observed in N1, suggesting an early compensatory mechanism was lost during progression [43,44]. While prior studies have connected miR-206 and LINC00261 to anti-angiogenic and tumor-suppressive roles in other malignancies [42,43], this is the first report to propose a direct involvement in metastatic PCa. In this context, the detectability of LINC00261, miR-206, and HIF1A in serum-derived liquid biopsies supports their prioritization as potential biomarker candidates, pending validation in larger independent cohorts.

Our findings show that early stage PCa (N0) is characterized by elevated levels of the tumor-suppressive miRNAs miR-206, miR-1-3p, miR-143-3p, and miR-145-5p, which target VEGFA, EGFR, SPHK1, and HIF1A [46,47,48,49]. Conversely, N1 displays decreased expression of these miRNAs and increased expression of pro-angiogenic genes (*HIF1A*, *CXCR4*, *VEGFA*, and *EGFR*) [41,51,52], suggesting transcriptional reprogramming during disease progression.

Nonetheless, our findings are preliminary and remain exploratory. Although relative expression differences have been confirmed, functional assays to demonstrate direct causality are still lacking. Sample size was representative but limited, and validation in cell-based and in vivo models, including studies with miRNA mimics/inhibitors and lncRNA knockdown or overexpression, will be essential to confirm these mechanisms. Extending these analyses to CRPC-derived organoids, xenografts, and prospective validation across independent patient cohorts, particularly those stratified by metastatic status and response to anti-angiogenic therapy, will be critical to assess the diagnostic and therapeutic potential of the identified axes will also help evaluate clinical relevance. Several screens have identified miRNA-lncRNA axes involved in metastasis and therapy resistance using high-throughput screening technologies, enabling the systematic identification of miRNAs associated with aggressive PCa phenotypes and stem-like features [57,58,59]. Altogether, these insights underscore the need for an integrated understanding of miRNA regulatory networks and their interactions with the broader molecular ecosystem of PCa. Given their multifaceted roles in angiogenesis, metastasis, and stem cell regulation, miRNAs are promising candidates for the development of novel diagnostic biomarkers and targeted therapeutic strategies to overcome resistance and prevent disease progression.

This study represents a preliminary but integrative approach for characterizing the transcriptomic regulatory networks involved in angiogenesis and metastasis within the context of CRPC. By leveraging publicly available RNA-seq datasets and predictive interaction platforms, we identified novel co-regulatory circuits involving miRNAs, lncRNAs, and mRNAs that may contribute to the modulation of angiogenic signaling and tumor dissemination and offer valuable insights into the molecular interplay between ncRNAs and pro-angiogenic gene expression, which must be validated in well-controlled experimental models. Despite these limitations, our findings provide a foundational framework for understanding the non-coding RNA-mediated regulation of angiogenesis in advanced PCa. Future studies should incorporate systematic network centrality analyses, as hubs such as the LINC00261–miR-206–HIF1A axis may represent crucial points of therapeutic intervention and may guide future efforts to develop RNA-based biomarkers and targeted therapeutic strategies.

## 5. Conclusions

This study presents a preliminary integrative framework for characterizing the transcriptomic regulatory networks involved in angiogenesis and metastasis in the context of CRPC. By leveraging publicly available RNA-seq datasets and predictive interaction platforms, we identified novel co-regulatory circuits involving miRNAs, lncRNAs, and mRNAs, which may play a role in modulating angiogenic signaling and tumor progression. Notably, our approach highlights the importance of regulatory network architecture, particularly the identification of highly connected axes, such as LINC00261–miR-206–HIF1A, which may act as critical hubs in angiogenesis control. Although the biological relevance of these interactions requires validation in well-controlled in vitro and in vivo models, our findings lay the groundwork for future studies aimed at translating these insights into RNA-based biomarkers and targeted therapies for advanced PCa.

## Data Availability

The original data presented in the study are openly available from 19 metastatic and 19 PCa samples (GSE117674) downloaded from the Gene Expression Omnibus (GEO) database (https://www.ncbi.nlm.nih.gov/geo/) (accessed on 28 May 2025). The target mRNAs of the differentially expressed miRNAs involved in the negative regulation of angiogenesis (GO:0016525) and positive regulation of angiogenesis (GO:0045766) were retrieved from the ENCORI database (https://rnasysu.com/encori/) (accessed on 5 June 2025). TCGA-PRAD data, including clinical stratification for N0 and N1 stages and miRNA/mRNA expression profiles, were downloaded from the UCSC Xena platform (http://xena.ucsc.edu/) (accessed on 16 June 2025). RNA-seq data for lncRNA expression from an experimental model of angiogenesis inhibition in CRPC C4-2 cells (GSE172205) were downloaded and analyzed using DESeq2. All bioinformatics analyses and regulatory network visualizations (including miRNA-lncRNA interactions) were conducted using publicly available databases, such as miRNet (https://www.mirnet.ca/) (accessed on 25 June 2025). Further data regarding these analyses, including interaction details, are available in the public domain and can be accessed through the respective platforms.

## Supplementary Materials

The following supporting information can be downloaded at https://www.mdpi.com/article/10.3390/biotech15010012/s1, Figure S1: Co-regulation network between miRNAs and lncRNAs associated with positive and negative regulation of angiogenesis; Figure S2: Anti-angiogenic transcriptomic signature: lncRNA–miRNA–mRNA regulatory network and lncRNA expression comparison between Angio (+) and Angio (−); Table S1: Primer sequences for qRT-PCR of lncRNA, miRNAs and target gene analyzed in this study.

## Abbreviations

The following abbreviations are used in this manuscript:

PCa	Prostate Cancer
CRPC	Castration-Resistant Prostate Cancer
GEO	Gene Expression Omnibus
miRNA	MicroRNA
lncRNA	Long Non-Coding RNA
VEGF	Vascular Endothelial Growth Factor
HUVECs	Human Umbilical Vein Endothelial Cells
mTORC1	Mechanistic Target of Rapamycin Complex 1
HIF-1α	Hypoxia-Inducible Factor 1-alpha
