# Supplementary material for: Transcriptomic Insights into lncRNA–miRNA–mRNA Networks Regulating Angiogenesis and Metastasis in Prostate Cancer
Source: BioTech (Basel). 2026 Feb 1;15(1):12. doi: 10.3390/biotech15010012 (PMC12921783; doi:10.3390/biotech15010012)
Supplement: Supplementary file 1 [file biotech-15-00012-s001.zip › Table S1.pdf]

Table S1. Primer sequences for qRT-PCR of lncRNA, miRNAs and target gene analyzed in this study.

Name	Type	Primer sequence
LINC00261 PMID: 39552712	lnCRNA	F: 5′- AAGACCAGCTCAACCATCGC -3′<br>R: 5′- TGCCATTTCCTGTGAATTGATGA-3′
hsa-miR-206 PMID: 38069061	miRNA	F: 5′- CTTCCCGAGGCCACATGCTT -3′<br>R: 5′- CACTTGCCGAAACCACACACT -3′
HIF1A PMID: 40530158	Axis target gene	F: 5′- GAAAGCGCAAGTCCTCAAAG -3′<br>R: 5′- TGGGTAGGAGATGGAGATGC 3′
snRNU6 PMID: **40004509**	Normalization	F: 5′- CTCGCTTCGGCAGCACA -3′<br>R: 5′-AACGCTTCACGAATTTGCGT -3′

F: Forward oligonucleotide. R: reverse oligonucleotide
