# Supplementary figures and images for: Transcriptomic Insights into lncRNA–miRNA–mRNA Networks Regulating Angiogenesis and Metastasis in Prostate Cancer
Source: BioTech (Basel). 2026 Feb 1;15(1):12. doi: 10.3390/biotech15010012 (PMC12921783; doi:10.3390/biotech15010012)

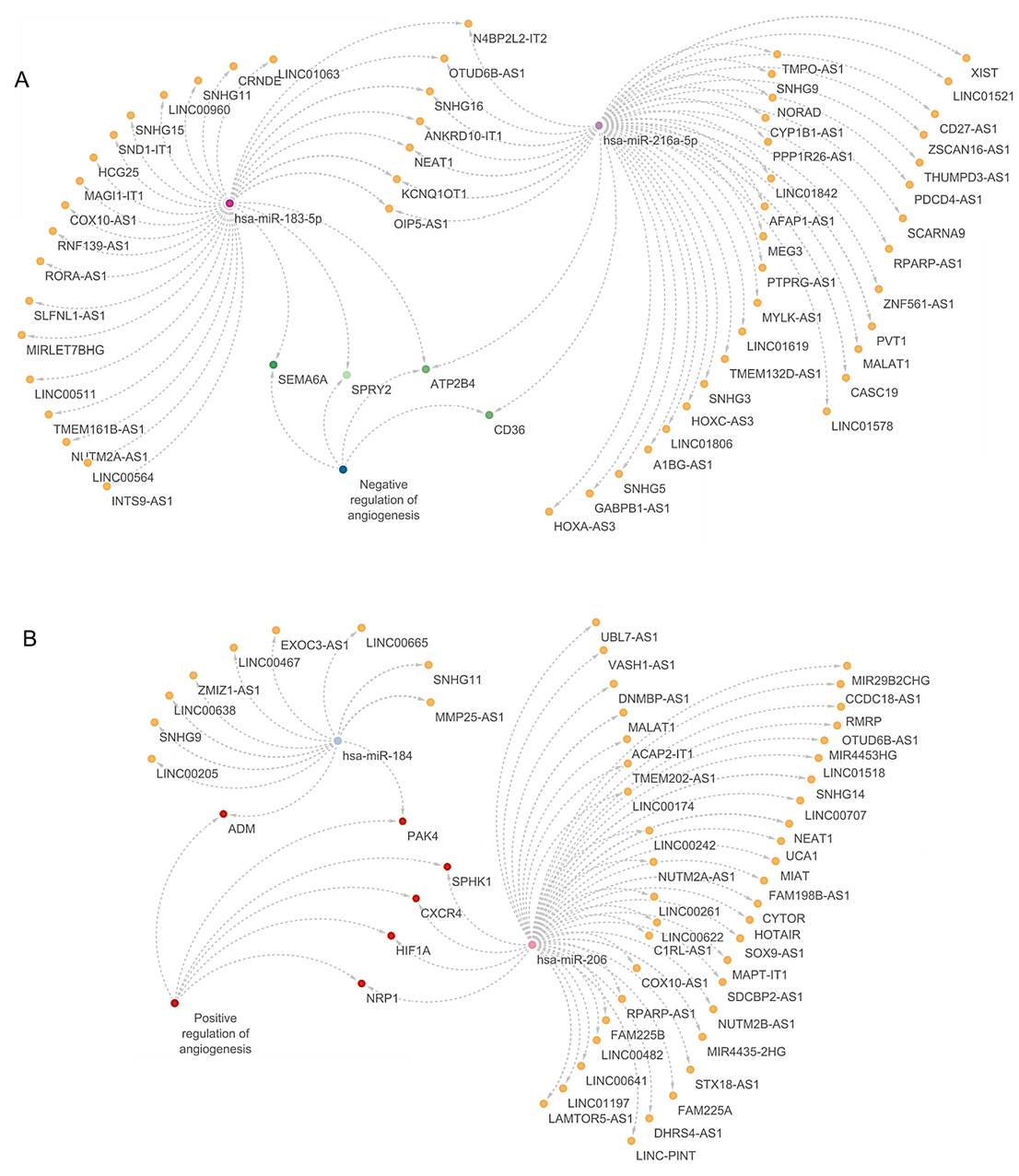

Supplement: Supplementary file 1 [file biotech-15-00012-s001.zip › Figure S1.jpg]

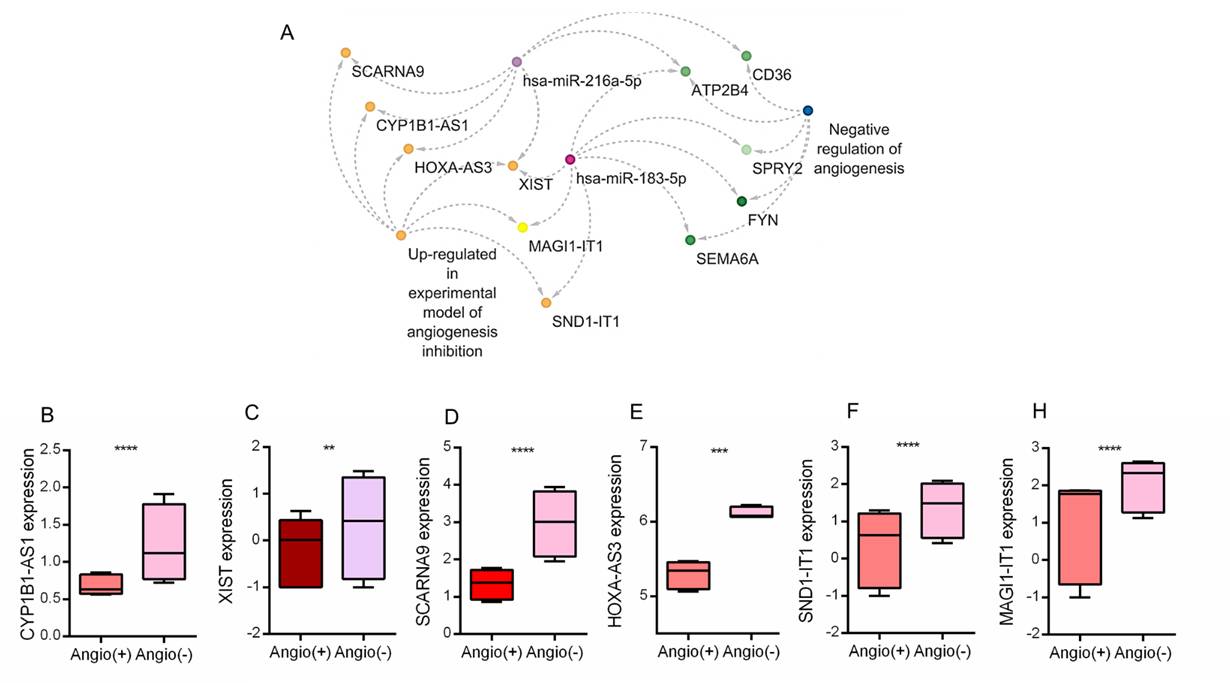

Supplement: Supplementary file 1 [file biotech-15-00012-s001.zip › Figure S2.jpg]
